# Earlier Is Better? Timing of Adductor Canal Block for Arthroscopic Knee Surgery under General Anesthesia: A Retrospective Cohort Study

**DOI:** 10.3390/ijerph18083945

**Published:** 2021-04-09

**Authors:** Shao-Chun Wu, Chih-Yi Hsu, Hsiao-Feng Lu, Chih-Chun Chen, Shao-Yun Hou, Yan-Yuen Poon

**Affiliations:** Department of Anesthesiology, Kaohsiung Chang Gung Memorial Hospital and Chang Gung University College of Medicine, Kaohsiung City 83301, Taiwan; shaochunwu@gmail.com (S.-C.W.); hsuchiyi@cgmh.org.tw (C.-Y.H.); maple@cgmh.org.tw (H.-F.L.); b9702062@cgmh.org.tw (C.-C.C.); walkings@cgmh.org.tw (S.-Y.H.)

**Keywords:** adductor canal block, anesthetic consumption, hemodynamic stability

## Abstract

The adductor canal block (ACB) is effective for treating postoperative pain during arthroscopic knee surgery, but its impact on anesthesia course and the optimal administration timing are unknown. This retrospective study addressed these questions. The aim of this study was to compare the effects of preoperative ACB and postoperative ACB on anesthesia course and postoperative recovery. We allocated 215 adult patients who underwent arthroscopic knee surgery under sevoflurane anesthesia between January 2019 and December 2019 to three groups. Group **A** received general anesthesia without ACB, Group **B** received ACB before general anesthesia induction, and Group **C** received ACB in the post-anesthesia recovery unit (PACU). Group **B** consumed significantly less sevoflurane (0.19 mL/kg/h) and milligram morphine equivalents (0.08 MME) intraoperatively than Groups **A** (0.22 mL/kg/h; 0.10 MME, respectively) and **C** (0.22 mL/kg/h; 0.09 MME, respectively). Groups **B** and **C** had lower visual analogue scale (VAS) scores upon PACU discharge than Group **A**. Dynamic, but not at-rest VAS scores, were significantly higher in Group **A**. Opioid consumption was similar in the ward, but Group **A** requested more intravenous parecoxib for pain relief. Length of hospital stay was similar. Thus, preoperative ACB reduced the amount of volatile anesthetic required and maintained stable hemodynamics intraoperatively. Preoperative or postoperative ACB improved postoperative pain control. Consequently, preoperative ACB is optimal for intraoperative stress suppression and postoperative pain control.

## 1. Introduction

Arthroscopy, i.e., inspection of a joint cavity, was initiated in the early 20th century by Professor Kenji Takagi [1] (1888–1963) and a Swiss surgeon, Dr. Eugene Bircher [2] (1882–1956). Due to the outbreak of the Second World War, arthroscopic knee surgery was not well-known in the orthopedic community until the late 1960s. With the evolution of newly designed instruments and techniques for arthroscopic surgeries, meticulous joint surgeries have become possible in contemporary orthopedic surgery.

Knee pain is one of the early signs of pathological change in joint cartilage, ligaments, meniscus or bone. Conservative treatment such as, intra-articular local anesthetic infiltration, oxygen-ozone therapy or hyaluronic acid [3], focal muscle vibration [4,5] is the usual treatment for these patients. Knee arthroscopy is a minimally invasive procedure that could reveal possible causes of knee pain and provide surgical repair of structural change in cartilage, ligament, meniscus or bone. Arthroscopic knee surgery [6] is one of the major surgeries in sports medicine and is particularly suitable for repairing torn cartilage [7], torn meniscus [8], or bone spurs [9]. While surgical wound size is smaller and tissue destruction is far less than that in conventional open knee surgeries, a substantial number of patients experience moderate to severe postoperative pain after arthroscopic knee surgeries [10,11] and improper management of postoperative pain may hinder rehabilitation [12] or delay early mobilization.

A variety of modalities for pain control have been proposed and used to alleviate postoperative pain after knee joint surgery. Systemic opioids have been used for postoperative pain control for decades; however, the immediate risk of respiratory depression or the long-term risks of opioid abuse, addiction, and overdose remain major concerns of surgeons [13]. Intra-articular injection of local anesthetics [14], morphine [15,16], or local anesthetics combined with morphine [17] has been used to alleviate postsurgical pain in knee surgeries; however, its efficacy has been questioned [18,19,20,21]. With great advances in ultra-sound technologies in recent years, ultrasound-guided peripheral nerve block has broadened its use in regional anesthesia and postoperative pain control [22].

The adductor canal block (ACB) is generally considered to be an effective analgesic technique for knee surgeries [23,24] because it offers satisfactory postoperative pain relief while preserving quadriceps strength [25,26]. The effectiveness of ACB reported in most studies [24,27] was assessed by means of resting and dynamic pain scores, time to first analgesic request, cumulative 24 h opioid consumption, ambulatory distance, length of hospital stay, and patient satisfaction. While ACB plays an indisputable role in postoperative pain control in knee surgery, there are two hitherto insufficiently addressed issues. First, to what extent does preoperative ACB affect the usual practice of general anesthesia? Second, does the timing of ACB administration, preoperatively or postoperatively, affect patient outcomes? The goal of the present study was to answer these two questions through a comprehensive review of pre-anesthesia, anesthesia, post-anesthesia recovery unit (PACU), and postoperative visit records of patients who underwent arthroscopic knee surgery under general anesthesia.

## 2. Materials and Methods

This study was approved by the Institutional Review Board of Kaohsiung Chang Gung Memorial Hospital (IRB number: 202100194B0). The Board waived the need to obtain informed consent because of the retrospective nature of the study. All methods were performed in accordance with the relevant guidelines and regulations. Anesthesia records of patients who underwent arthroscopic knee surgery from January 2019 to December 2019 were retrieved from the hospital’s database. Exclusion criteria included day surgery, spinal anesthesia, desflurane anesthesia, anesthesia without bispectral index monitor (BIS)-guidance, and records with missing data.

Patients were segregated into three groups. Group **A** patients received general anesthesia only; Group **B** patients received preoperative ACB, before induction of general anesthesia; Group **C** patients received postoperative ACB at the PACU, upon request. As a standard practice in our hospital, general anesthesia was induced with propofol (1–2 mg/kg). The use of rocuronium (1 mg/kg), cis-atracurium (0.2 mg/kg), alfentanil (10 mcg/kg), and sevoflurane (1‒1.3 MAC) depends on the anesthesiologists’ preferences, and a fresh gas flow of 50% oxygen with air was maintained at 1 L/min. The BIS score was maintained in the range of 40‒60 during anesthesia. ACB was performed using an ultrasound-guided technique with a total injection volume of 21 mL, which was a mixture of 10 mL 0.5% levobupivacaine, 5 mL 2% lidocaine, 5 mL normal saline, and 1 mL dexamethasone.

Options for postoperative pain control included intravenous opioids, intravenous parecoxib, oral acetaminophen, or ACB. Obtaining informed consent for ACB is compulsory in our hospital. ACB is usually performed before induction of general anesthesia, as in Group **B** patients, while postoperative ACB is performed upon the request of patients, as in Group **C** patients. We collected all perioperative data during surgery, including vital signs, administered drugs, sevoflurane consumption, and data during the stay in the PACU. We also included data from routine daily postoperative visits, which were performed by well-trained nurse anesthetists within 24 h of surgery. The visual analog scale (VAS, 0–10) was used to assess postoperative pain response in this study. VAS was also used as an indicator of the efficacy of the pain treatment modality in the ward. VAS scores were taken when patients were at rest or in a dynamic state.

### Statistical Analysis

Categorical variables, such as sex, ASA physical status, postoperative nausea and vomiting (PONV) risk, and incidence of comorbidities were compared using the chi-square or Fisher’s exact test. Data are presented as the raw numbers or percentages. Continuous numeric data were tested using one-way analysis of variance with Bonferroni correction. Numerical data are presented as the median (25%–75%). Statistical significance was set at *p* < 0.05.

## 3. Results

**Figure 1:**
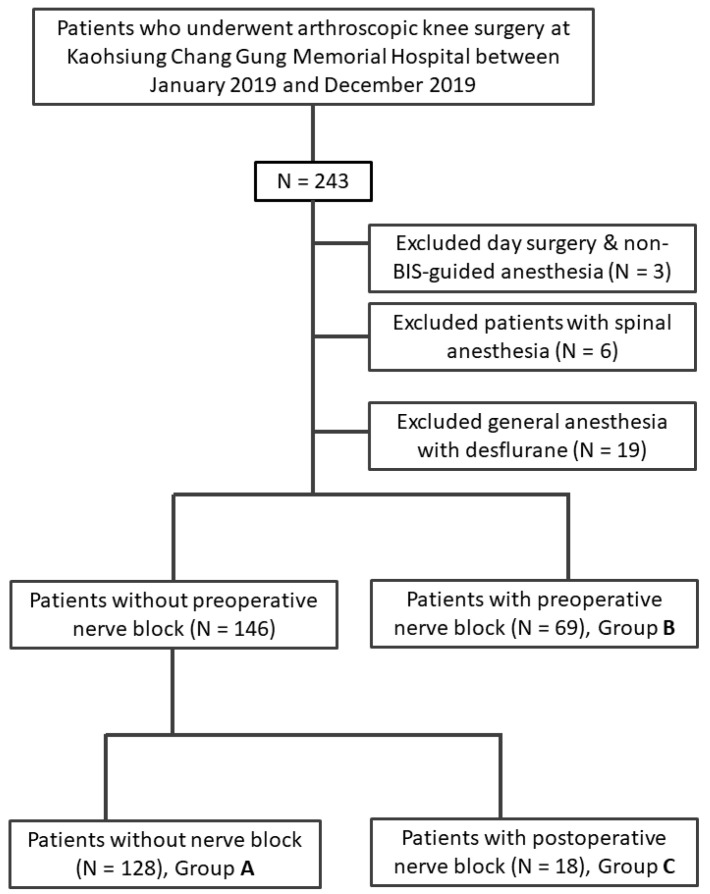
Flow chart of allocation of patients underwent arthroscopic knee surgery into Group **A** (non-ACB), Group **B** (preoperative ACB) and Group **C** (postoperative ACB).

A total of 243 anesthesia records of patients who underwent arthroscopic knee surgery were retrieved from the hospital’s database. We included 215 patients after exclusions. We deliberately excluded patients under desflurane anesthesia because the number of cases was too small. Patients were segregated into Groups **A** (*n* = 128), **B** (*n* = 69), and **C** (*n* = 18), as shown in Figure 1. Table 1 summarizes the demographic characteristics of the patients.

There were no significant differences in sex distribution, age, body weight, ASA physical status, anesthesia time, risk stratification of PONV, or comorbidities among the three groups (Table 1). The temporary change in sevoflurane concentration was significantly higher in Group **A** and Group **C** than in Group **B** in the first hour of operation (2.50%, 2.65%, and 2.20%, respectively; Table 2). The hourly consumption of sevoflurane was significantly higher in patients without preoperative ACB (Group **A** and in Group **C**) than in those with preoperative ACB (Group **B**), i.e., 0.22 mL/kg/h, and 0.22 mL/kg/h, 0.19 mL/kg/h, respectively (Table 2). For intraoperative opioid consumption, patients in Groups **A** and **C** had significantly higher morphine milligram equivalent (MME) consumption than those in Group **B** (0.10 MME, 0.09 MME, and 0.08 MME, respectively) (Table 2). Baseline systolic blood pressure was similar among the three groups. Our results revealed a significantly higher occurrence of surgery-induced hypertension in the early phase of surgery in patients in Groups **A** and **C** than in those in Group **B** (25.8%, 27.8%, and 11.6%, respectively) (Table 2). Intraoperative hypertension was defined as an increase in systolic blood pressure of 30% or more over baseline systolic blood pressure.

Our results showed that a significantly higher VAS score was recorded in Group **A** than in Groups **B** and **C** (3.0, 1.0, and 1.0, respectively), at PACU discharge (Table 3). For opioid consumption in the PACU, there was no significant difference in MME consumption among the three groups, although a slightly higher MME was recorded in Groups **A** and **C** (Table 3). There was no significant difference in the occurrence of PONV among the three groups during the PACU stay.

There was no significant difference in VAS scores at rest among the groups, while a significantly higher VAS score was recorded in Group **A** (3.0) than in Group **B** (2.0) and Group **C** (1.5) in the dynamic state (Table 3). Opioid consumption was similar among the three groups (Table 3). More patients in Group **A** required intravenous parecoxib than those in Groups **B** and **C** (35.4%, 13.0%, and 16.7%, respectively). For oral acetaminophen, the requirements were similar among the three groups (Table 3). The risk of postoperative side effects was similar among the three groups, although more patients experienced PONV in Group **A**. No postoperative nerve injury was reported in this study. The length of hospital stay after surgery was similar among the three groups. High satisfaction scores were recorded in all three groups in this study (Table 3).

## 4. Discussion

In this study, we investigated the effect of ACB on the course of anesthesia in patients undergoing arthroscopic knee surgery, and evaluated the appropriate timing of delivery of the block. We found that preoperative ACB reduced the amount of volatile anesthetic required and maintained stable hemodynamics intraoperatively. Both preoperative and postoperative ACB improved postoperative pain control.

Peripheral nerve block (PNB) has gained in popularity in recent years because it can provide reliable and effective postoperative pain control in many surgeries [28]. The first successful nerve block was reported on 6 December 1884, by Richard John Hall [29]. In the early years, target nerve injection was confirmed by assessing paresthesia. This was gradually replaced by the nerve stimulation technique, which further increased the success of PNB. With the introduction of high-resolution sonography in recent years, direct visualization of nerves and accurate injection of local anesthetics in the vicinity of target nerves has become possible [30]. Ultrasound-guided nerve blocks not only improve the quality of the nerve block, but also decrease the dose of local anesthetics injected and hence pose a lower risk of local anesthetic toxicity [31].

Arthroscopic knee surgery is frequently performed in sports medicine, and good postoperative pain control makes early rehabilitation of knee joints possible. Early rehabilitation is important for all patients. The return to sporting activity is particularly important for athletes [32]. ACB is generally considered to be the first-choice postoperative pain control modality in knee surgeries, because it can provide fairly good pain relief without compromising motor function [24,25,26]. We found that preoperative ACB affected general anesthesia in that a lower concentration of sevoflurane was recorded in the early phase of surgery, when the surgical stimulus was assumed to be intense. Our previous study [33] showed that the first hour of sevoflurane anesthesia reflects the phase of uptake of volatile anesthetics when a high concentration gradient exists between alveolar and exogenous gas supply. This may explain why a higher concentration of sevoflurane was required in patients without preoperative ACB to suppress the surgical stimulus during the early phase of surgery when the uptake of the volatile anesthetic is still underway. The effectiveness of preoperative ACB in lowering the surgical stimulus during surgery was further supported by lower sevoflurane consumption and a lower opioid requirement in patients who had received the ACB. All patients in the study underwent BIS monitoring to ensure that anesthesia levels were adequate.

Our study revealed two important findings: First, preoperative ACB reduced the consumption of volatile anesthetic that may have an impact on PONV. Previous reports indicated that volatile anesthetic is a strong anesthesia-related predictor of PONV [34]. It is reasonable to speculate that avoidance of excessive volatile anesthetics may reduce the number of patients experiencing PONV. Our study supported this speculation, in that fewer patients with preoperative ACB had PONV, although the difference was not statistically significant. Our results also revealed that preoperative ACB avoided excessive perturbation of cardiovascular functions by a high concentration of inhalational anesthetic, in an attempt to suppress surgical stress. Previous reports have shown that a prolonged fluctuation of mean blood pressure of more than 35% from baseline is significantly associated with the occurrence of a postoperative stroke [35,36]. The second important implication of this study was that preoperative ACB could facilitate a stable hemodynamic state during surgery, which is particularly important in patients with impaired cardiovascular function.

In terms of appropriate timing of performing ACB, our findings indicate that preoperative ACB is preferable to postoperative ACB for reducing sevoflurane consumption or intraoperative opioid consumption. However, the efficacy of ACB in alleviating postoperative pain was similar in both preoperative and postoperative ACB groups upon discharge from the PACU, while a higher VAS score was recorded in Group **A**. An interesting finding was that 12.3% of patients (Group **C**/Group **A** + Group **C**, 18/146) undergoing arthroscopic knee surgery requested rescue ACB in the PACU. The relatively small number of patients who requested rescue ACB may reflect the fact that arthroscopic knee surgeries may not cause severe pain in the majority of patients, as previously reported [27]. A lower rest VAS score or dynamic VAS score was recorded in the ward in patients who had received either pre- or postoperative ACB. It is interesting to note that, despite a time delay of more than 2 h between Groups **B** and **C** in terms of receiving the ACB, the analgesic effect was similarly effective in the ward. Supporting evidence for the analgesic benefit of ACB was that more patients in Group **A** required rescue analgesics and parecoxib for pain control in the ward. Another benefit of ACB may be that, among patients who had received ACB, fewer patients experienced PONV, although this was not statistically significant.

## 5. Conclusions

We concluded that preoperative ACB could modify the anesthesia course by reducing the concentration and consumption of volatile anesthetic during surgery. A tendency toward reducing PONV was also observed in patients with preoperative ACB. For postoperative pain control, both preoperative ACB and postoperative ACB were effective in alleviating postoperative pain. Thus, we recommend that preoperative ACB should be considered in patients undergoing arthroscopic knee surgery because it could provide good postoperative pain control and facilitate a stable hemodynamic state for surgery, without excessive use of inhalational anesthetics.

## Figures and Tables

**Table 1:** Demographic characteristics of patients who underwent arthroscopic knee surgery under bispectral index-guided sevoflurane anesthesia, without adductor canal block (ACB) (Group **A**), with preoperative ACB (Group **B**), and with postoperative ACB (Group **C**).

	Group A (*n* = 128)	Group B (*n* = 69)	Group C (*n* = 18)	*p* Value
Gender (Male/Female)	65/63	32/37	7/11	0.59
Age (years)	44.0 (30.5–57.5)	44.5 (27.0–58.8)	47.5 (38.8–52.0)	0.767
Body weight	69.0 (61.0–81.0)	69.5 (58.3–81.5)	70.0 (59.5–82.0)	0.997
ASA				
I	18 (14.1%)	12 (17.4%)	1 (5.6%)	0.23
II	97 (75.8%)	54 (78.2%)	17 (94.4%)	
III	13 (10.1%)	3 (4.4%)	0 (0%)	
Anesthesia time (hour)	1.88 (1.57–2.50)	2.18 (1.67–2.49)	2.35 (1.85–2.81)	0.177
PONV risk				
Low risk	34 (26.4%)	18 (26.5%)	5 (27.8%)	0.984
High risk	95 (73.6%)	50 (73.5%)	13 (72.2%)	
Arthroscopic surgery				0.368
Cruciate ligament	18 (14.1%)	17 (24.6%)	5 (27.8%)	
Medial/Lateral ligament	1 (0.8%)	1 (1.4%)	1 (5.6%)	
Meniscus	49 (38.3%)	22 (31.9%)	6 (33.3%)	
Synovial/Articular shaving	60 (46.9%)	29 (42.0%)	6 (33.3%)	
Comorbidity				
Hypertension	26 (20.3%)	12 (17.4%)	4 (22.2%)	0.874
Diabetes Mellitus	7 (5.5%)	5 (7.2%)	2 (11.1%)	0.621
COPD	2 (1.6%)	0 (0.0%)	0 (0.0%)	0.51
CAD	1 (0.8%)	1 (1.1%)	0 (0.0%)	0.811
CHF	0 (0.0%)	0 (0.0%)	0 (0.0%)	-
CVD	1 (0.8%)	0(0.0%)	0 (0.0%)	0.715
ESRD	0 (0.0%)	0 (0.0%)	0 (0.0%)	-

Numeric values are expressed as median (IQR) or numbers (%). PONV: postoperative nausea and vomiting, COPD: chronic obstructive pulmonary disease, ESRD: end-stage renal disease, CAD: coronary artery disease, CHF: congestive heart failure, CVD: cerebral vascular disease.

**Table 2:** Intraoperative presentations of patients who underwent knee arthroscopic surgery under bispectral index-guided sevoflurane anesthesia without adductor canal block (ACB) (Group **A**), with preoperative ACB (Group **B**), and with postoperative ACB (Group **C**).

	Group A (*n* = 128)	Group B (*n* = 69)	Group C (*n* = 18)	*p* Value
Mean sevoflurane concentration (%) in 1st hour	2.50 (2.20–2.90)	2.20 (1.9–2.7)	2.65(2.50–2.80)	0.004
Sevoflurane consumption (ml/kg/h)	0.22 (0.18–0.27)	0.19 (0.15–0.25)	0.22 (0.21–0.26)	0.005
Intraoperative fluid given (ml/kg)	2.26 (1.74–2.80)	2.08 (1.74–2.47)	1.97 (1.71–2.40)	0.175
Baseline systolic blood pressure (mmHg)	136 (124–153)	142 (130–155)	134 (119–146)	0.477
Patients with intraoperative hypertensive response	33 (25.8%)	8 (11.6%)	5 (27.8%)	0.042
Intraoperative opioid consumption (MME)	0.10 (0.08–0.13)	0.08 (0.06–0.11)	0.09 (0.07–0.12)	0.001

Numeric values are expressed as median (IQR) or numbers (%). MME, morphine milligram equivalent.

**Table 3:** Postoperative presentations of patients who underwent knee arthroscopic surgery under bispectral index-guided sevoflurane anesthesia without adductor canal block (Group **A**), with preoperative ACB (Group **B**), and with postoperative ACB (Group **C**).

	Group A (*n* = 128)	Group B (*n* = 69)	Group C (*n* = 18)	*p* Value
VAS at PACU	3.0 (2.0–4.0)	1.0 (1.0–2.0)	1.0 (1.0–2.0)	<0.001
VAS at ward				
Rest	1.00 (1.00–2.00)	1.00 (0.00–1.00)	1.0 (0.00–1.00)	0.932
Dynamic	3.00 (2.00–4.00)	2.00 (1.00–2.00)	1.50 (1.00–2.00)	<0.001
**Number of patients required analgesics at ward**				
Parecoxib (intravenous)	45 (35.1%)	9 (13.0%)	3 (16.7%)	0.001
NSAID (oral)	64 (50%)	27 (39.1%)	10 (55.6%)	0.258
**Opioid consumption at ward**				
MME	0.00 (0.00–0.38)	0.00 (0.00–0.13)	0.00 (0.00–0.09)	0.137
**Number of patients with postoperative side effects**				
Headache	1 (0.8%)	1 (1.4%)	0 (0.0%)	0.763
Nausea	5 (3.9%)	2 (2.9%)	1 (5.6%)	0.861
Vomiting	10 (7.8%)	4 (5.8%)	1 (5.6%)	0.840
Dizziness	9 (7.0%)	5 (5.6%)	1 (5.6%)	0.966
Length of stay after surgery (day)	3.00 (2.00–4.38)	3.00 (2.00–4.00)	3.50 (2.50–4.38)	0.505
Patient satisfaction (1–5)	5.00 (4.00–5.00)	5.00 (4.00–5.00)	5.00 (4.00–5.00)	0.955

All values are shown as median (interquartile range) or number and percent. ACB: adductor canal block, PACU: post anesthesia room, MME: milligram morphine equivalents, VAS: Visual analogue scale.

## Data Availability

Data available in a publicly accessible repository.
